# Vagus nerve stimulation as an add-on therapy in patients with epilepsy: a prospective, multicenter, real-world study in China

**DOI:** 10.1186/s42494-026-00272-4

**Published:** 2026-08-11

**Authors:** Chenyang Zhao, Enhui Zhang, Hesheng Zhang, Raowei Yan, Zhijun Le, Yujie Chen, Wenyu Liu, Weixi Xiong, Xintong Wu, Dong Zhou

**Affiliations:** Departments of Neurology, West China Hospital, Sichuan University, Chengdu, Sichuan 610041 People’s Republic of China

**Keywords:** Outcome, Neurostimulation, Follow-up, VNS

## Abstract

**Background:**

Vagus nerve stimulation (VNS) has been proven as an effective and safe adjunct therapy for epilepsy, but real-world evidence is limited. This study aimed to evaluate outcomes of VNS and its cumulative effect through a prospective, multicenter, real-world survey in China, with dynamic follow-up.

**Methods:**

A total of 83 sites in China participated and 124 epilepsy patients enrolled. Visits were scheduled at 1, 3, 6, 9, and 12 months after VNS. Primary outcomes included seizure response rate (≥ 50% frequency reduction) and seizure-free rate. Secondary outcomes assessed changes in the 31-item Quality of Life in Epilepsy Inventory (QOILE-31) score, overall anti-seizure medication (ASM) load, and adverse events.

**Results:**

The responder rates were 35.5%, 41.6%, 62.2%, 64.2%, and 76.6% at 1, 3, 6, 9, and 12 months after VNS, respectively. Seizure-free rates were 2.4%, 2.7%, 9.2%, 8.6%, and 10.9% at 1, 3, 6, 9, and 12 months, respectively. No significant differences were observed in overall ASM load between baseline and any follow-up visit. Significant improvements were noted in QOLIE-31 from baseline to each follow-up visit, with mean (± SD) score improvements as follows: 1 month (2.93 ± 8.65), 3 months (4.27 ± 9.84), 6 months (4.69 ± 11.5), 9 months (6.68 ± 12.5), and 12 months (9.32 ± 13.8). A significant trend toward improved seizure outcomes and change in quality of life was observed with longer treatment durations. Adverse events occurred in 34 patients (27.4%).

**Conclusions:**

VNS is an effective and safe adjunctive treatment for epilepsy, reducing seizure frequency and significantly improving quality of life, with a cumulative effect over time.

**Supplementary Information:**

The online version contains supplementary material available at 10.1186/s42494-026-00272-4.

## Background

Epilepsy is a common neurological disorder, affecting an estimated 70 million people globally [1]. Approximately one-third of people with epilepsy who are treated with two appropriately chosen, administered, and well-tolerated anti-seizure medications (ASMs), either as monotherapy or in combination, still struggle to control their seizures [2]. For such patients, early pre-surgical evaluation is recommended. However, resection or disconnection of the seizure-onset zone is only feasible for certain patients. When surgery is not applicable, neuromodulation therapy is recommended [3].

Vagus nerve stimulation (VNS) is a neuromodulation therapy that stimulates the left cervical vagus nerve, sending afferent signals to the nucleus tractus solitarius, which then activates the locus coeruleus and other brain regions [4]. Several long-term open-label studies have reported the efficacy and safety of VNS as a palliative therapy for epilepsy patients [5, 6]. It was approved by the Food and Drug Administration in 1997 as an adjunctive therapy for patients with pharmacoresistant epilepsy. In China, VNS was gradually introduced into clinical practice starting in 2008 as a palliative therapy for drug-resistant epilepsy, although it remained underutilized initially [7]. The domestic VNS product in China completed its clinical trial registration in 2014 and began being used in clinical settings in 2016. Following the registration and application of domestic VNS, the number of VNS treatments saw rapid growth from 2015 to 2018. Currently, over 140 hospitals in China offer VNS therapy, and nearly 5,000 epilepsy patients have received this treatment [8]. Xu et al. reported that 52 adult patients underwent VNS implantation for the treatment of refractory epilepsy between May 2016 and May 2017 at five Epilepsy Centers, with a 66.0% responder rate at the 1-year follow-up [9]. Shan et al. retrospectively evaluated the clinical outcome of 45 consecutive patients with drug-resistant epilepsy undergoing VNS implantation in a single center between November 2016 and August 2021 and reported a 48.9% responder rate [10]. However, there is still a lack of large-scale cohort studies with nationwide coverage involving more than 100 patients in China regarding the application of VNS. And it remains unclear whether longer follow-up periods are associated with a significantly greater trend toward seizure freedom or an increased responder rate [3].

Here, we present the one-year outcomes from this nationwide real-world study of epilepsy patients receiving add-on VNS therapy to: (1) evaluate the clinical outcomes of VNS as a palliative therapy in drug-resistant epilepsy patients, including changes in seizure burden, quality of life, and overall ASM load; (2) assess the safety of VNS application; and (3) determine, through dynamic follow-up, whether the effects of VNS on seizure control and quality of life accumulate over time.

## Methods

### Participants

This is a multicenter, prospective, real-world study of the application of VNS for adult patients with epilepsy in China. A total of 83 sites in China participated in this study, and patients included in this study underwent VNS device implantation surgery between January 2022 and December 2024. This study was approved by the ethics committee of West China Hospital (2022 − 1847), and informed consent was obtained from all patients. Eligible participants were patients diagnosed with drug-resistant epilepsy according to the 2010 International League Against Epilepsy (ILAE) criteria [2]. Exclusion criteria included: (1) age < 18 years or > 75 years, (2) failure to complete at least one month of follow-up after implantation.

### Study design

Presurgical evaluation, inclusion and exclusion criteria of VNS therapy, implantation of the VNS device (PINS, Beijing, China), adjustment of ASMs, and setting of VNS parameters were all carried out by each respective hospital, following a standardized procedure [8, 11] (details see supplementary information methods part).

Patients who met the eligibility criteria underwent a 12-week prospective baseline period to collect baseline data, including seizure frequency, the 31-item Quality of Life in Epilepsy Inventory (QOLIE-31), and other demographic and clinical information (Table 1). Following VNS implantation, study visits were scheduled at 1, 3, 6, 9, and 12 months to gather follow-up data. All data collection was carried out using standardized Case Report Forms (CRFs). ASM load was defined as the total dosage rate relative to the standard dose for all ASMs used, as follows [12]:

$$\rm{Overall\,ASM\,load} = \sum (Dosage / Standard\,dosage)$$

The standard dose was referenced from the World Health Organization (WHO) website (https://atcddd.fhi.no/atc_ddd_index/).

### Endpoints

The primary endpoint was the change in seizure frequency from baseline, expressed as the percentage change from baseline frequency. This included seizure-free, a ≥ 75% but < 100% reduction (75% reduction), a ≥ 50% but < 75% reduction (50% reduction), a ≥ 25% but < 50% reduction (25% reduction), a ≥ 0% but < 25% reduction (no change), and an increase in frequency. Patients were considered responders if they had a reduction of ≥ 50% in seizure frequency compared to baseline; conversely, they were classified as non-responders. Secondary endpoints included the change in score of QOLIE-31, overall ASM load, and adverse events.

### Statistical analysis

All statistical analyses were performed using SPSS 26.0 software, with *P*-values < 0.05 considered statistically significant. Continuous variables were presented as mean ± standard deviation (SD) or median (IQR), depending on the data distribution. Categorical variables were described using absolute frequencies and percentages. The Wilcoxon signed-rank test or Student’s paired *t*-test was used for continuous variables, depending on their distribution. The Chi-Square Test or Fisher’s exact test was used for the comparison of categorical variables. Correlation analyses were conducted between changes in QOLIE-31 scores and clinical characteristics prior to VNS implantation, including sex, age at implantation, age at seizure onset, epilepsy duration, seizure frequency, number of ASMs, overall ASM load, etiology, seizure types, and comorbidities, as well as whether the patient was a responder at the last visit. These analyses were performed using Pearson correlation, Spearman correlation, or Kendall’s tau-b correlation analysis.

## Results

### Demographic and baseline clinical features

A total of 124 patients (77 males and 47 females) with a mean age of 28.3 ± 11.2 (median 24.5, range 18–67) years were enrolled in this study. The mean age at seizure onset was 14.5 ± 11.3 (median 12.0, range 1–61) years, the mean duration of epilepsy was 14.8 ± 10.2 (median 13.1, range 0.3–50.8) years. Details are presented in Table 1.

**Table 1:** Patient characteristics

Variables	Number (%) or mean±SD
**Sex**	
Male	77 (62.1%)
Female	47 (37.9%)
**Age at VNS implantation (years)**	
Mean±SD	28.3±11.2
**Age at seizure onset (years)**	
Mean±SD	14.5±11.3
**Epilepsy duration prior to VNS (years)**	
Mean±SD	14.8±10.2
**Seizure frequency prior to VNS, no./m**	
Mean±SD	13.4±39.6
**ASMs no. prior to VNS**	
0	3 (2.4%)
1	28 (22.6%)
2	45 (36.3%)
3	27 (21.8%)
4	13 (10.5%)
5	6 (4.8%)
6	2 (1.6%)
**Overall ASMs load prior to VNS**	
Mean±SD	1.57±1.02
**Etiology**	
Structural	81 (65.3%)
Genetic	6 (4.8%)
Infectious	13 (10.5%)
Metabolic	6 (4.8%)
Immune	2 (1.6%)
Unknown	16 (12.9%)
**Seizure types**	
Focal onset	69 (55.6%)
FBTCS (yes / no)	51 (73.9%) / 18 (26.1%)
Generalized onset	47 (37.9%)
Focal and generalized onset	1 (0.8%)
Unknown	7 (5.6%)
**Comorbidities**	
No comorbidities	56 (45.2%)
Anxiety	23 (18.5%)
Depression	13 (10.5%)
Migraine	1 (0.8%)
Mental retardation	22 (17.7%)
Language development delay	12 (9.7%)
Cerebrovascular diseases	1 (0.8%)
Cognitive impairment	34 (27.4%)
Developmental encephalopathy	5 (3.9%)
Others	5 (3.9%)

SD, standard deviation; VNS, vagus nerve stimulation; no./m, number per month; ASMs, anti-seizure medications; FBTCS, focal to bilateral tonic-clonic seizure

### Seizure outcomes after VNS

**Fig. 1:**
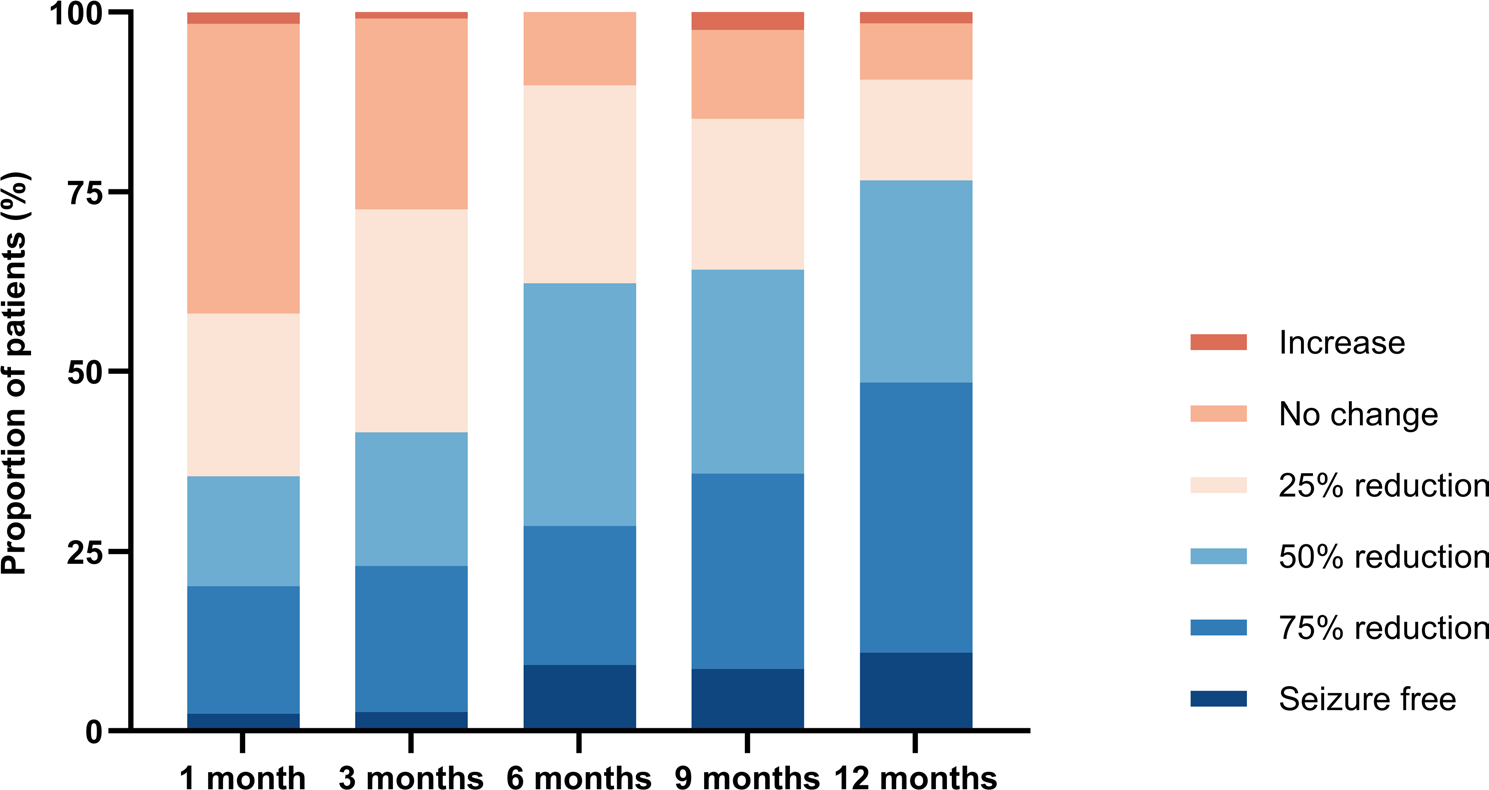
The proportion of patients with different seizure changes according to duration of VNS therapy

A ≥ 50% seizure reduction was achieved in 35.5%, 41.6%, 62.2%, 64.2%, and 76.6% of patients at 1, 3, 6, 9, and 12 months after VNS, respectively, with the proportion of responders increasing over time (*P* < 0.05, Table 2, Table S1, Fig. 1). For seizure-free patients, there were 3 (2.4%), 3 (2.7%), 9 (9.2%), 7 (8.6%), and 7 (10.9%) at 1, 3, 6, 9, and 12 months after VNS, respectively, showing a significant trend toward increased seizure freedom with longer follow-up periods (*P* < 0.05, Table S1-2). No significant differences in overall ASM load were observed between baseline and each follow-up visit (Table S1).

**Table 2:** Responder rate for each follow-up compared to baseline

	Responder (%)	Non-responder (%)	χ^2^	*P*
Baseline	0 (0.0)	124 (100.0)	-	-
1 month	44 (35.5)	80 (64.5)	53.5	< 0.001
3 months	47 (41.6)	66 (58.4)	64.3	< 0.001
6 months	61 (62.2)	37 (37.8)	106.4	< 0.001
9 months	52 (64.2)	29 (35.8)	106.7	< 0.001
12 months	49 (76.6)	15 (23.4)	128.4	< 0.001

### QOLIE-31 outcomes after VNS

**Fig. 2:**
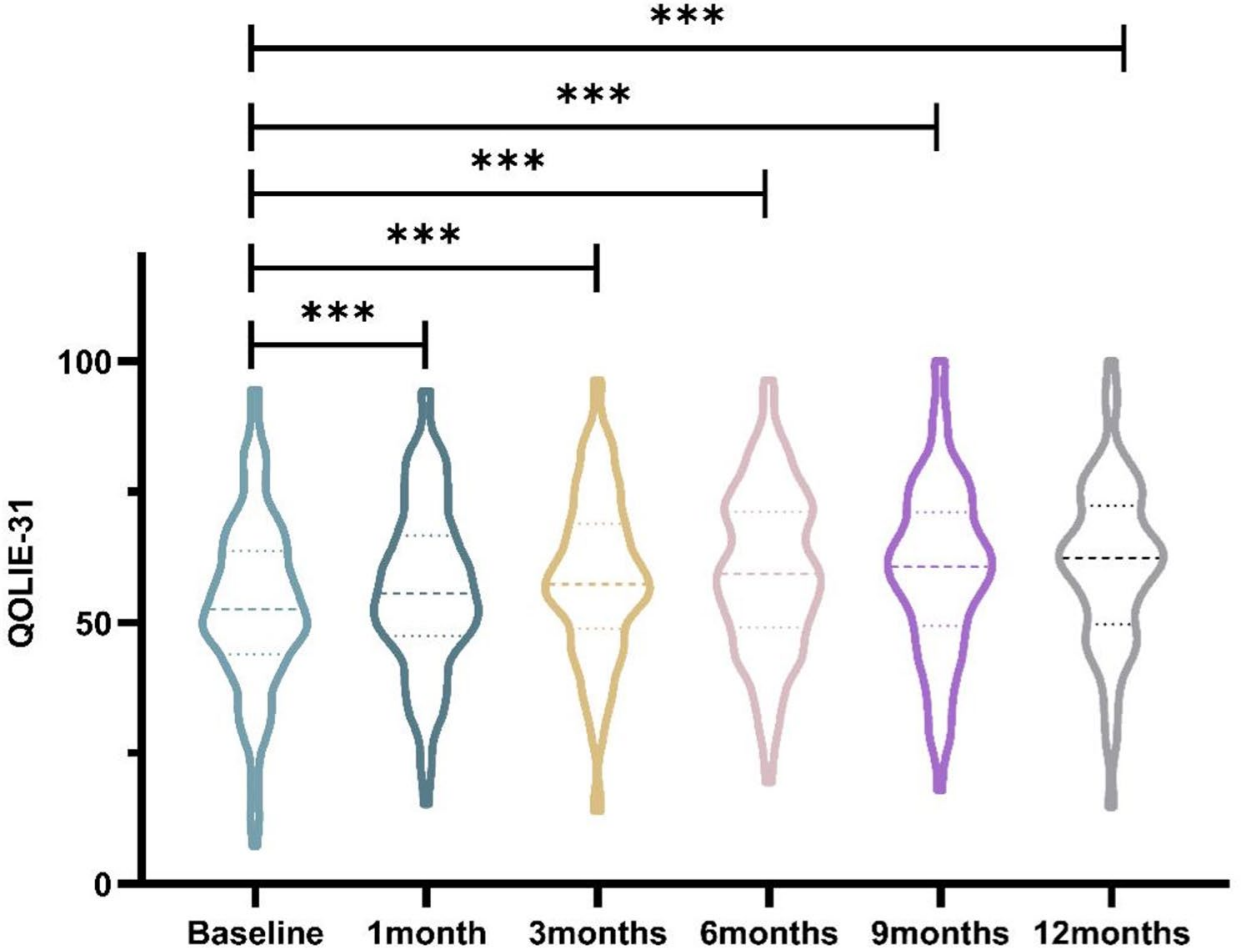
Change in QOLIE-31 overall score from baseline to 1, 3, 6, 9, and 12 months after VNS. *** *P* < 0.001

Significant improvements were observed in QOLIE-31 scores between baseline and each follow-up visit, with increasing benefits over time (*P* < 0.05, Table S2). The mean (± SD) score improvements were as follows: 1 month (2.93 ± 8.65), 3 months (4.27 ± 9.84), 6 months (4.69 ± 11.5), 9 months (6.68 ± 12.5), and 12 months (9.32 ± 13.8). Details are shown in Fig. 2 and Table S1.

### Safety

At least one adverse event was reported in 34 patients (27.4%). Among these, the most commonly reported adverse events were voice changes or hoarseness and cough, accounting for 76.5% and 32.4% of all adverse events, respectively (Table 3). All documented adverse events occurred at the start of stimulation phase. These events were generally transient and reversible.

**Table 3:** Adverse events after VNS

Adverse events	Number (%)
No	90 (72.6%)
Yes	34 (27.4%)
Voice change or hoarseness	26 (21.0%)
Cough	11 (8.9%)
Nausea	2 (1.6%)
Headache	1 (0.8%)
Infection	2 (1.6%)
Hematoma	2 (1.6%)
Localized neck pain	1 (0.8%)

VNS, vagus nerve stimulation

### Correlation analysis

**Fig. 3:**
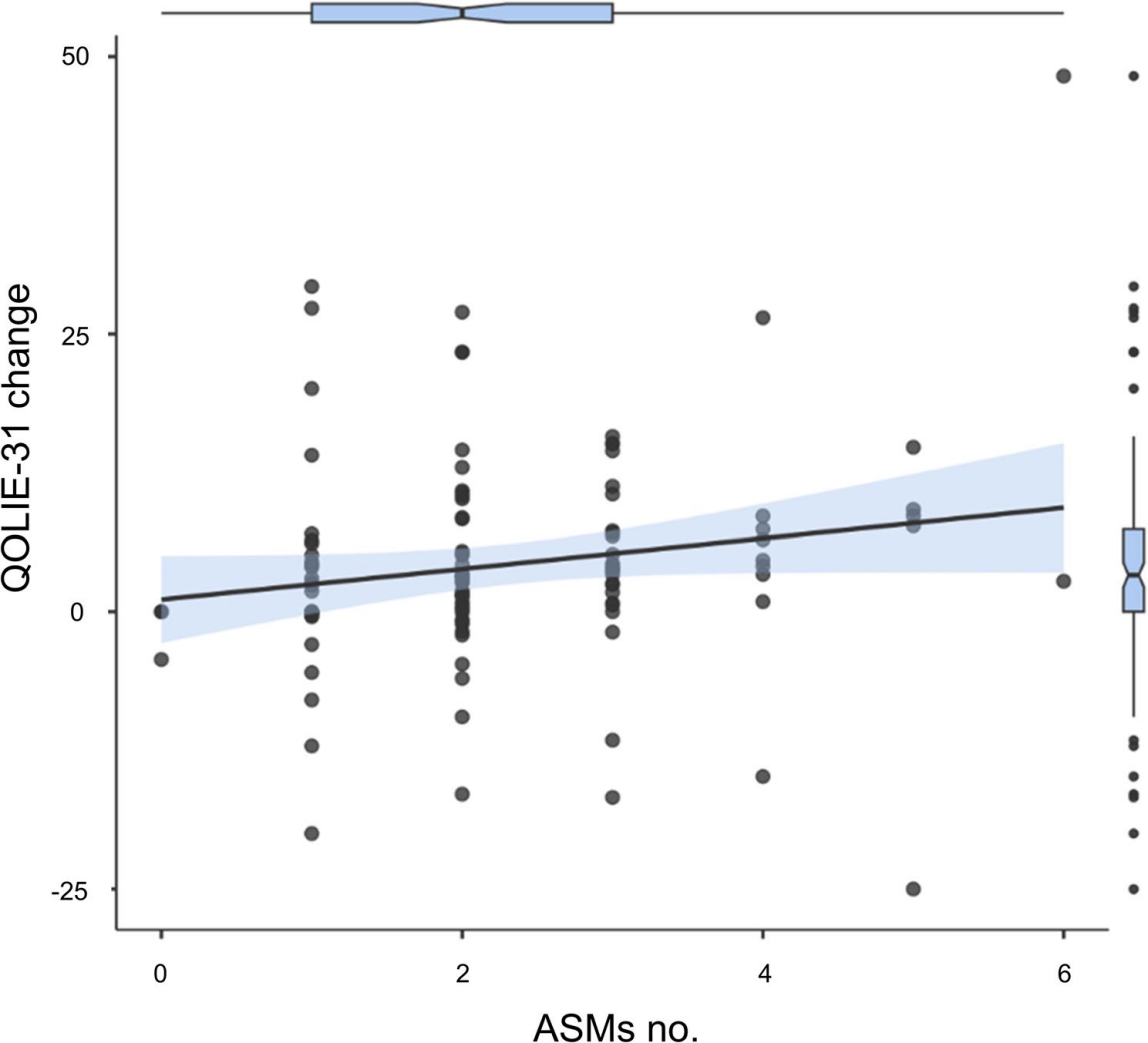
Positive correlation between the number of ASMs prior to VNS and change in QOLIE-31 scores at 3 months post-implantation. The scatter plot illustrates the positive correlation between the number of ASMs prior to VNS and the change in QOLIE-31 scores at 3 months after implantation (*rs* = 0.243, *P* = 0.010)

There were no significant correlations between sex, age at VNS implantation, age at seizure onset, epilepsy duration prior to VNS, seizure frequency before VNS, overall ASM load prior to VNS, etiology, seizure types, comorbidities, and the change in QOLIE-31 scores at 1, 3, 6, 9, and 12 months following VNS implementation (*P* > 0.05). However, the number of ASMs prior to VNS showed a positive correlation with the change in QOLIE-31 scores at 3 months after implantation (*r* = 0.243, *P* = 0.010, Table S3, Fig. 3). In addition, whether the patient was a responder at the last visit positively correlated with change of QOLIE-31 scores at 3 (*r* = 0.167, *P* = 0.031), 6 (*r* = 0.168, *P* = 0.044), 9 (*r* = 0.200, *P* = 0.030), and 12 (*r* = 0.242, *P* = 0.019) months following VNS implementation (Table S3).

## Discussion

As the first nationwide, multicenter, real-world study of VNS in China, this investigation prospectively evaluated the efficacy and safety of VNS as a palliative therapy for patients with drug-resistant epilepsy through a dynamic one-year follow-up. We reported a mean responder rate of 56.0% and a mean seizure-free rate of 6.8% following VNS therapy. Our results were comparable to those of other studies. A recent systematic review, which included 11 studies, reported a mean responder rate of 56.9%, ranging from 48.9% to 83.0% [13]. A multicenter study from Japan reported mean seizure freedom rates of 6.4%, and mean responder rates of 52.9%, respectively [12]. Another multicenter study from Belgium reported a seizure freedom rate of 9% and a responder rate of 59%, with a mean follow-up of 44 months [14]. A recent meta-analysis found that the proportion of individuals experiencing seizure freedom at the last follow-up was 3.3% (based on data pooled from 10 case series), and the proportion of responders was 42.7% (based on data pooled from 19 case series) [3]. Our data revealed a significant cumulative effect of VNS therapy, with the responder rate and seizure-free rate reaching their highest values at the final follow-up, at 76.6% and 10.9%, respectively. Previous studies [5, 10, 12, 15, 16] have also demonstrated that VNS leads to a gradual improvement in seizure reduction over time. However, a meta-analysis study, which pooled data from 4 randomized controlled trials and 19 case series to investigate the application of VNS, proposed that there was a non-significant trend toward greater seizure freedom or proportion of responders with longer follow-up periods [3]. In our study, we directly compared the proportion of seizure freedom and responders during dynamic follow-up and showed a significant cumulative effect of VNS.

A slight, non-significant increasing trend in mean ASM load from baseline to follow-up was observed in our study, despite significant improvements in seizure outcomes and quality of life. This observation aligns with previous studies that also reported increases in both ASM load [12] and the number of ASM used [6]. One plausible explanation is that in patients who achieve substantial, yet incomplete, seizure reduction, clinicians may cautiously optimize or even slightly increase the existing medication regimen in pursuit of seizure freedom.

Our study reported a substantial improvement in quality of life following VNS therapy, with a mean improvement of 5.58 in QOLIE-31 scores. Consistent with our findings, several studies have demonstrated the impact of VNS on the quality of life of epilepsy patients. The PuLsE study showed a significant difference between the VNS plus best medical practice (BMP) group and the BMP-only group, with patients in the VNS plus BMP group experiencing a greater improvement in quality of life [5]. Klinkenberg et al. reported a significant mean increase of 6.56 in the QOLIE-89 score after 6 months of VNS treatment [17]. Additionally, two prospective cohort studies from Japan [12] and Korea [18] also confirmed the role of VNS in improving the quality of life of epilepsy patients. Similar to the seizure outcomes, the improvement in quality of life gradually increased over time, reaching its peak at the end of the follow-up period, consistent with previous studies [5]. However, it is important to note that as the follow-up duration increased, the dropout rate also rose, which may introduce a potential selection bias. The study found a positive correlation between the number of ASMs used prior to VNS and the improvement in quality of life following VNS therapy. In other words, the greater the number of ASMs taken before VNS, the more significant the improvement in quality of life after VNS treatment. Previous studies confirmed that adverse events related to ASMs are a key factor influencing the quality of life in epilepsy patients [19, 20]. Also, the responder of VNS has a more significant improvement in QOLIE-31 scores.

No severe or life-threatening adverse events were reported in our study, nor were there any events that led to the removal of the VNS device. The overall incidence of adverse events was 27.4%, with the most commonly reported events being voice changes or hoarseness (21.0%) and cough (8.9%). Compared to other reports, our results are generally consistent and may even show a slight improvement. Shan et al. reported that among 45 VNS patients, the overall incidence of adverse events was 57.8%, with the most common being hoarseness (22.2%), surgical site discomfort (11.1%), and coughing (8.9%) [10]. The overall incidence of adverse events in other studies was 45.8% [3] and 71.4%, respectively [21]. A meta-analysis revealed that the most common complications of VNS were hoarseness (37.2%), followed by cough (14.2%) and throat pain (9.6%) [3].

Our study has several limitations. Firstly, the dosing of ASMs and the adjustment of parameters during VNS therapy for each patient were not standardized and were left to the discretion of their respective physicians. However, as a real-world study, this approach reflects typical clinical practice, where treatment decisions are individualized based on each patient’s specific needs and circumstances. Notably, ASM adjustments were made in a subset of patients (37 of 124). Despite these individual regimen changes, the overall ASM load for the entire cohort showed no statistically significant difference from baseline at any follow-up visit. Secondly, there was a loss to follow-up at every visit, which may introduce bias into the results. Lastly, our study had a maximum follow-up period of 12 months. Future research will involve longer-term, rigorous follow-up to assess the long-term effects of VNS on seizure outcomes and quality of life, as well as its cumulative effects.

## Conclusions

This prospective, multicenter, real-world study with continuous follow-up affirmed that VNS is an effective adjunctive and palliative treatment and safe for patients with drug-resistant epilepsy in a highly heterogeneous cohort. Patients who are not suitable for conventional resective surgery can benefit from VNS, which not only reduces seizure frequency but also significantly improves quality of life, with evidence showing a cumulative effect of this efficacy over time.

## Supplementary Information

Below is the link to the electronic supplementary material.

Supplementary Material 1

## Data Availability

All data are available from the corresponding author upon reasonable request.

## Abbreviations

ASMs: Anti-seizure medications
VNS: Vagus nerve stimulation
QOLIE-31: 31-item Quality of Life in Epilepsy Inventory
CRFs: Case Report Forms
WHO: World Health Organization
SD: Standard deviation
FBTCS: Focal to bilateral tonic-clonic seizure
BMP: Best medical practice
