# Supplementary material for: Vagus nerve stimulation as an add-on therapy in patients with epilepsy: a prospective, multicenter, real-world study in China
Source: Acta Epileptol. 2026 Aug 11;8:30. doi: 10.1186/s42494-026-00272-4 (PMC13459777; doi:10.1186/s42494-026-00272-4)
Supplement: Supplementary file 1 [file 42494_2026_272_MOESM1_ESM.docx]

Supplementary materials

**Contents**

[Supplementary methods	2](#_Toc221138761)

[1. Inclusion and exclusion criteria for VNS	2](#_Toc221138762)

[2. Implantation and setting of VNS parameters	2](#_Toc221138763)

[Supplementary results	4](#_Toc221138764)

[Table S1. Seizure outcomes, QOLIE-31 changes, and overall ASMs load changes at each visit after VNS implementation	4](#_Toc221138765)

[Table S2 Comparisons of responder rate, seizure free rate, and QOLIE-31 changes between visits	5](#_Toc221138766)

[Table S3. Correlations between the change of QOLIE-31 and the clinical variables	6](#_Toc221138767)

[References	7](#_Toc221138768)

# **Supplementary methods**

1. Inclusion and exclusion criteria for VNS

Patients were eligible if they met all of the following criteria:

(1) Diagnosis of drug‑resistant epilepsy according to the 2010 International League Against Epilepsy (ILAE) definition;

(2) No treatable etiology identified, or failure of targeted treatment for a known etiology;

(3) Unsuitability for resective surgery or documented failure of prior epilepsy surgery.

Patients were excluded if they presented with any of the following:

(1) History of bilateral vagus nerve injury or transection;

(2) Active infection at the planned implantation site;

(3) High risk of implant rejection;

(4) Poor general medical condition precluding safe surgical tolerance.

2. Implantation and setting of VNS parameters

The VNS therapy was delivered using a domestically manufactured vagus nerve stimulation system (PINS., Beijing, China). This device received market approval from China’s National Medical Products Administration (NMPA) in 2015. Prior studies have demonstrated that the therapeutic efficacy and safety profile of this device are comparable to those of earlier-generation VNS systems used internationally[1-3]. The implantation procedure, lead configuration, and device programming were performed in strict accordance with established clinical standards and safety guidelines[4, 5], ensuring alignment with the conventional VNS therapy paradigm.

The general titration protocol followed by participating centers is summarized below. The typical titration protocol commenced with low-intensity initial settings (0.2-0.5 mA, 30 Hz, 250/500 μs, 30 s ON/5 min OFF) 1–2 weeks after implantation. Provided the patient tolerated stimulation well, the output current was generally increased by 0.2-0.5 mA every two weeks during the first three months. Thereafter, from three months post-implant onward, parameters were reviewed and optimized every three months. Optimization followed one of two common strategies: either progressively increasing the current toward the maximum tolerated level before adjusting the duty cycle, or prioritizing an increase in the duty cycle before modifying the current.

# **Supplementary results**

Table S1. Seizure outcomes, QOLIE-31 changes, and overall ASMs load changes at each visit after VNS implementation

|  | Baseline  N=124 | 1 month  N=124 | 3 months  N=113 | 6 months  N=98 | 9 months  N=81 | 12 months  N=64 |
| --- | --- | --- | --- | --- | --- | --- |
| **Seizure outcome N (%)** | | | | | | |
| Seizure free | 0 (0.0) | 3 (2.4) | 3 (2.7) | 9 (9.2) | 7 (8.6) | 7 (10.9) |
| 75% reduction | 0 (0.0) | 22 (17.7) | 23 (20.4) | 19 (19.4) | 22 (27.2) | 24 (37.5) |
| 50% reduction | 0 (0.0) | 19 (15.3) | 21 (18.6) | 33 (33.7) | 23 (28.4) | 18 (28.1) |
| 25% reduction | 0 (0.0) | 28 (22.6) | 35 (31.0) | 27 (27.6) | 17 (21.0) | 9 (14.1) |
| No change | 124 (100.0) | 50 (40.3) | 30 (26.6) | 10 (10.2) | 10 (12.4) | 5 (7.8) |
| Increase | 0 (0.0) | 2 (1.6) | 1 (0.9) | 0 (0.0) | 2 (2.5) | 1 (1.6) |
| *P* | - | <0.001* | <0.001* | <0.001* | <0.001* | <0.001* |
| **QOLIE-31 change (mean ± SD)** | | | | | | |
| QOLIE-31 | 53.4±16.3 | 56.3±15.8 | 58.7±15.7 | 59.5±15.7 | 60.1±16.4 | 61.0±15.8 |
| *P* | - | <0.001* | <0.001* | <0.001* | <0.001* | <0.001* |
| **ASMs change** | | | | | | |
| ASMs no.  (median, range) | 2 (0,6) | 2 (0,6) | 2 (0,6) | 2 (0,6) | 2 (0,6) | 2 (0,6) |
| ASMs load (mean ± SD) | 1.57±1.02 | 1.70±1.39 | 1.75±1.58 | 1.73±1.42 | 1.80±1.52 | 1.84±1.66 |
| *P* | - | 0.333 | 0.443 | 0.169 | 0.056 | 0.072 |

QOLIE-31, 31-item Quality of Life in Epilepsy Inventory; ASMs, antiseizure medications, no. number; * *P* < 0.05

Table S2 Comparisons of responder rate, seizure free rate, and QOLIE-31 changes between visits

|  | Responder rate  Statistical value (*P*) | Seizure free rate  Statistical value (*P*) | QOLIE-31 change  Statistical value (*P*) |
| --- | --- | --- | --- |
| 1 month vs 3 months | 2.88 (0.090) ^a^ | 0.00 (1.000) ^a^ | 4033 (<0.001) * ^b^ |
| 1 month vs 6 months | 21.1 (<0.001) * ^a^ | 4.50 (0.034) * ^a^ | 1634 (0.024) * ^b^ |
| 1 month vs 9 months | 18.2 (<0.001) * ^a^ | 3.57 (0.059) ^a^ | 870 (<0.001) * ^b^ |
| 1 month vs 12 months | 26.1 (<0.001) * ^a^ | 3.57 (0.059) ^a^ | 375 (<0.001) * ^b^ |
| 3 months vs 6 months | 18.2 (<0.001) * ^a^ | 6.00 (0.014) * ^a^ | 1722 (0.327) ^b^ |
| 3 months vs 9 months | 13.5 (<0.001) * ^a^ | 3.57 (0.059) ^a^ | 850 (0.002) * ^b^ |
| 3 months vs 12 months | 23.1 (<0.001) * ^a^ | 6.00 (0.014) * ^a^ | 63.0 (<0.001) * ^c^ |
| 6 months vs 9 months | 0.250 (0.617) ^a^ | 0.667 (0.414) ^a^ | 658 (<0.001) * ^b^ |
| 6 months vs 12 months | 9.00 (0.003) * ^a^ | 5.00 (0.025) * ^a^ | 225 (<0.001) * ^b^ |
| 9 months vs 12 months | 8.00 (0.005) * ^a^ | 3.00 (0.083) ^a^ | 312 0.001) * ^b^ |

QOLIE-31, 31-item Quality of Life in Epilepsy Inventory

^a^ Chi-Square Test

^b^ Wilcoxon signed-rank test

^c^ Student’s paired t-test

* *P* < 0.05

Table S3. Correlations between the change of QOLIE-31 and the clinical variables

|  | **Clinical variables** | 1 month | |  | 3 months | |  | 6 months | |  | 9 months | |  | 12 months | |
| --- | --- | --- | --- | --- | --- | --- | --- | --- | --- | --- | --- | --- | --- | --- | --- |
|  |  | *r* | *P* value^a^ |  | *r* | *P* value^a^ |  | *r* | *P* value^a^ |  | *r* | *P* value^a^ |  | *r* | *P* value^a^ |
|  | Sex^b^ | -0.057 | 0.444 |  | 0.021 | 0.787 |  | -0.068 | 0.414 |  | -0.039 | 0.669 |  | -0.022 | 0.835 |
|  | Age at VNS implantation^b^ | 0.058 | 0.523 |  | -0.072 | 0.451 |  | -0.052 | 0.613 |  | -0.137 | 0.223 |  | -0.070 | 0.584 |
|  | Age at seizure onset^b^ | 0.065 | 0.473 |  | 0.107 | 0.261 |  | 0.048 | 0.644 |  | 0.023 | 0.836 |  | 0.075 | 0.558 |
|  | Epilepsy duration prior to VNS^b^ | -0.021 | 0.818 |  | -0.140 | 0.146 |  | -0.036 | 0.728 |  | -0.139 | 0.222 |  | -0.123 | 0.341 |
|  | Seizure frequency before VNS^b^ | 0.026 | 0.773 |  | 0.173 | 0.067 |  | 0.121 | 0.234 |  | 0.115 | 0.305 |  | 0.088 | 0.489 |
|  | Number of ASMs before VNS^b^ | 0.088 | 0.333 |  | 0.243* | 0.010 |  | 0.137 | 0.178 |  | 0.015 | 0.891 |  | -0.063 | 0.618 |
|  | Overall ASM load prior to VNS^b^ | 0.022 | 0.807 |  | 0.117 | 0.219 |  | 0.067 | 0.513 |  | -0.020 | 0.858 |  | -0.045 | 0.721 |
|  | Etiology^b^ | 0.047 | 0.603 |  | -0.018 | 0.850 |  | -0.011 | 0.916 |  | 0.035 | 0.755 |  | -0.012 | 0.927 |
|  | Seizure types^b^ | 0.106 | 0.239 |  | -0.050 | 0.600 |  | -0.011 | 0.913 |  | -0.011 | 0.924 |  | 0.005 | 0.966 |
|  | Comorbidities^b^ | 0.102 | 0.258 |  | 0.131 | 0.167 |  | 0.048 | 0.636 |  | 0.041 | 0.715 |  | -0.102 | 0.421 |
|  | Responder at the last visit^c^ | 0.141 | 0.056 |  | 0.167* | 0.031 |  | 0.168* | 0.044 |  | 0.200* | 0.030 |  | 0.242* | 0.019 |

^a^ Statistical threshold was set at p<0.05, two tailed

^b^ Spearman correlation

^c^ Kendall’s tau-b correlation

* *P* < 0.05

QOLIE-31, 31-item Quality of Life in Epilepsy Inventory; VNS, vagus nerve stimulation; ASMs, antiseizure medication

# **References**

1. Tan B, Liu Y, Gong M, Meng F, Yang A, Zhang K, et al. Long-term efficacy and predictors of vagus nerve stimulation in drug-resistant epilepsy: a multicenter cohort study. Acta epileptologica. 2025;7(1):40. doi: 10.1186/s42494-025-00220-8.

2. Ma J, Wang Z, Cheng T, Hu Y, Qin X, Wang W, et al. A prediction model integrating synchronization biomarkers and clinical features to identify responders to vagus nerve stimulation among pediatric patients with drug-resistant epilepsy. CNS neuroscience & therapeutics. 2022;28(11):1838-48. doi: 10.1111/cns.13923.

3. Cheng TY, Hu Y, Qin X, Ma J, Zha D, Xie H, et al. A predictive model combining connectomics and entropy biomarkers to discriminate long-term vagus nerve stimulation efficacy for pediatric patients with drug-resistant epilepsy. CNS neuroscience & therapeutics. 2024;30(7):e14751. doi: 10.1111/cns.14751.

4. China Epilepsy Association. Standard Operating Procedure (Draft) for Vagus Nerve Stimulation in the Treatment of Epilepsy. Epilepsy Journal, 2025, 11(02): 96-99+120.

5. Wu Y, Zhou J, Guan YG, Meng FG, Ji TY. Chinese Expert Consensus on Vagus Nerve Stimulation for the Treatment of Drug-resistant Epilepsy. Epilepsy Journal. 2021; 7(03): 191-6.
